# GIF-2209, an Oxindole Derivative, Accelerates Melanogenesis and Melanosome Secretion via the Modification of Lysosomes in B16F10 Mouse Melanoma Cells

**DOI:** 10.3390/molecules27010177

**Published:** 2021-12-28

**Authors:** Miyu Watanabe, Kyoka Kawaguchi, Yusuke Nakamura, Kyoji Furuta, Hiroshi Takemori

**Affiliations:** Department of Chemistry and Biomolecular Science, Faculty of Engineering, Gifu University, 1-1 Yanagido, Gifu 501-1193, Japan; z4521088@edu.gifu-u.ac.jp (M.W.); a4521029@edu.gifu-u.ac.jp (K.K.); z4521060@edu.gifu-u.ac.jp (Y.N.); furuta@gifu-u.ac.jp (K.F.)

**Keywords:** oxindole, SU5205, melanogenesis, melanosome, tyrosinase, TYRP-1, lysosome, B16F10

## Abstract

Melanogenesis and melanosome secretion are regulated by several mechanisms. In this study, we found that the oxindole derivative GIF-2209 accelerated melanogenesis associated with the discrimination in the expression and intracellular distributions of two melanogenic enzymes, tyrosinase (TYR) and tyrosinase-related protein-1 (TYRP-1). GIF-2209 upregulated the expression of TYR via a microphthalmia transcription factor (MITF)-independent mechanism, leading to high expression of protein. In contrast, GIF-2209 did not alter the mRNA levels of TYRP-1 and suppressed its protein levels. GIF-2209 induced the dissociation of TYR from TYRP-1 but did not alter the association between TYR and CD63, a melanosome and lysosome marker. The protein levels of CD63 were also upregulated by GIF-2209. GIF-2209 induced lysosome expansion and redistribution in all areas of the cytosol, accompanied by autophagy acceleration (upregulation of LC3BII protein levels and downregulation of p62 protein levels). In addition, GIF-2209 stimulated the secretion of melanosomes containing high levels of TYR, TYRP-1, and CD63 proteins. The GIF-2209 mediated melanosome secretion was sensitive to the lysosome inhibitor chloroquine. These results suggest that GIF-2209 may activate lysosomal functions with *TYR* gene expression, while it accelerates melanosome secretion, which finally leads to the depletion of intracellular melanogenic enzyme, especially TYRP-1 protein.

## 1. Introduction

Melanosomes are classified as lysosome-related organelles in which melanin is synthesized to protect the skin from ultraviolet (UV) irradiation [1,2]. In response to cellular signaling pathways, for example, including cAMP and p38, melanogenic enzymes such as tyrosinase (TYR) and tyrosinase-related protein 1/2 (TYRP-1/2)] are synthesized and transferred to melanosomes in melanocytes [3]. When melanosomes mature via the accumulation of melanin, the melanosomes are secreted from melanocytes and incorporated into keratinocytes by several mechanisms, including pinocytosis and phagocytosis.

A number of small compounds that modulate melanogenesis have been reported and are categorized into several groups based on the mechanism of action, cellular signaling [4,5], transcription [6], translation [7], cellular trafficking [8], and degradation [9]. Post-translational modulators of melanogenic factors are often associated with intracellular trafficking and affect the maturation and fate of melanosomes and related organelles [10].

Tetraspanin CD63, also known as lysosomal-associated membrane protein 3 (LAMP3), is a marker of melanosomes and lysosomes [11]. Exosomes and some extracellular vesicles share organelles for their synthesis with melanosomes, and the exosomes are also enriched in CD63 protein on their surface [12]. The CD63 protein is supplied either as a newly synthesized protein via the trans-Golgi network or as an incorporated protein via endocytosis of exosomes and then transported to early endosomes. Finally, the CD63 protein then participates in vesicle transport systems, thus interacting with melanogenic and lysosomal enzymes and exosome factors, and is redistributed to melanosomes, lysosomes, and exosomes [13,14].

Lysosomes are organelles that digest cellular components to reuse them depending on the types of cellular demands [15]. When the cellular components deteriorate with age, they are transported to lysosomes and digested in a low-pH environment. Large components, including organelles, are wrapped with an extra membrane (autophagosome) and fused with lysosomes, leading to autolysosomes. Melanogenic enzymes are also targets of lysosomes [16], and the inhibition of lysosomal activities by chloroquine and other pH neutralizers results in the accumulation of target molecules, including TYR [8,17]. Similar to impairments in the TYR enzyme, some small compounds that inhibit TYR activity accelerate the recruitment of the TYR enzyme to lysosomes. Therefore, the suppression of melanogenesis by TYR inhibitors is often associated with lower levels of TYR protein [18].

However, it is known that lysosomal dysfunction ultimately impairs melanogenesis. The lysosome inhibitor chloroquine elevates the expression of TYR but suppresses melanogenesis [8]. Here, we report that GIF-2209, an oxindole derivative, upregulates melanogenesis. In mouse melanoma B16F10 cells, this compound had multiple effects, including the upregulation of TYR and CD63 mRNA(s) and protein(s), without affecting the levels of the melanogenic master transcriptional regulator microphthalmia transcription factor (MITF) or TYRP-1 mRNA(s). When the cells were treated with a high concentration of GIF-2209, TYRP-1 protein levels decreased. Although TYR is co-localized with TYRP-1 in control cells, GIF-2209 segregated TYR from TYRP-1 without affecting TYR-CD63 co-localization. Although lysosomes are localized mainly to perinuclear regions, GIF-2209 treatment resulted in lysosome enlargement and redistribution throughout the cytoplasm. GIF-2209 increased melanosome secretion, suggesting lysosomal activation may promote this process.

## 2. Results

### 2.1. Structure–Activity Relationship of Oxindole Derivatives

**Figure 1:**
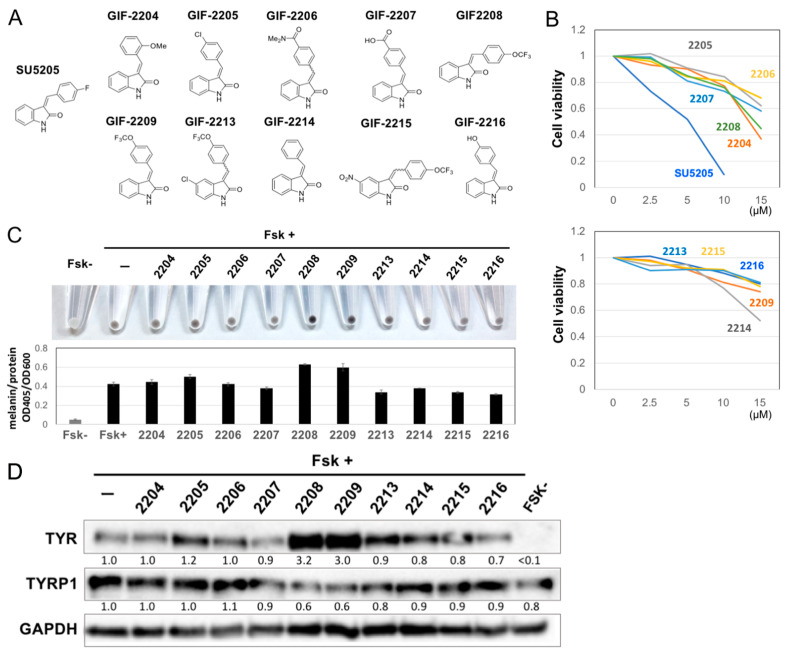
(**A**) Structures of oxindole derivatives (GIF compounds). (**B**) B16F10 cells were treated with compounds for 48 h. Cell viability was measured with WST-8. Means of duplicated experiments are shown. (**C**) B16F10 cells were treated with 5 µM of oxindole derivatives in the presence of 20 µM forskolin (Fsk) for 48 h. Melanin production in the B16F10 cells was visually evaluated by photo, and the melanin content was measured after alkaline lysis and normalized to protein content. *n* = 3. (**D**) Protein expression for TYR, TYRP-1, and GAPDH in B16F10 cells that had been treated as in (**C**) were examined by western blotting. The images are representative of triplicated experiments. The band intensity for TYR and TYRP1 was measured by ChemiDoc XRS+ and normalized to GAPDH intensity.

To identify new melanogenesis modulators, we screened 2000 compounds in the Molport chemical library and found SU5205 as a candidate melanogenesis accelerator [19]. SU5205, an oxindole derivative (Figure 1A), has been developed as a multi-kinase inhibitor for anticancer drugs and shows cellular toxicity at concentrations less than 10 μM (Figure 1B). We therefore synthesized 10 oxindole derivatives and evaluated their effects on B16F10 mouse melanoma cells.

We found that 5 µM of trifluoro derivatives, GIF-2208 and GIF-2209, accelerated melanogenesis induced by the cAMP elevator Fsk (Figure 1C). Modifications of the benzene ring of oxindole weakened the melanogenic acceleration activity (demonstrated by GIF-2213 and GIF-2215). Thus, GIF-2209 was selected as the melanogenic accelerator. Unexpectedly, we found that GIF-2209, as well as GIF-2208, upregulated TYR protein levels but downregulated TYRP-1 protein levels (Figure 1D). As the 4′-hydroxy type, GIF-2216, showed no or rather inhibitory effect on Fsk-induced melanogenesis, we used GIF-2216 as a control compound (we later realized that this decision was a mistake).

### 2.2. GIF-2209 Has Multiple Mechanisms of Action on Melanogenesis

**Figure 2:**
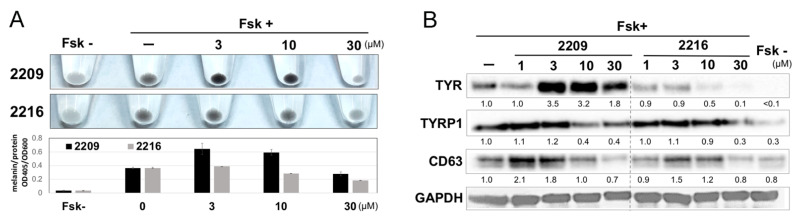
(**A**) B16F10 cells were treated with 20 µM Fsk and the indicated concentrations of GIF-2209 or GIF-2216 for 48 h. Cells were recovered in sample tubes and collected by centrifugation for the visual examination, and melanin content was measured after alkaline lysis and normalized to protein content. *n* = 3. **(B**) Western blot analyses. The images are representative of triplicate experiments. The band intensity for TYR, TYRP-1, and CD63 was measured by ChemiDoc XRS and normalized to GAPDH intensity.

To characterize GIF-2209 in detail, dose dependency was examined. GIF-2209 accelerated melanogenesis evaen at 3 μM (Figure 2A), accompanied by increased levels of TYR protein. In contrast, no significant difference in the TYRP-1 protein level was observed at 3 μM, whereas a decrease was observed at 10 μM, suggesting the presence of multiple regulatory mechanisms on GIF-2209-mediated melanogenesis modification. The existence of multiple mechanisms was also supported by the protein level of the lysosome and melanosome marker CD63, which was upregulated by GIF-2209 at 1 μM.

GIF-2216 also upregulated CD63 protein levels at 1 μM, while downregulating TYR protein levels at concentrations greater than 10 μM with no or small effects on TYRP-1 protein level.

### 2.3. GIF-2209 Upregulates TYR Transcription Independent of MITF Expression Level

**Figure 3:**
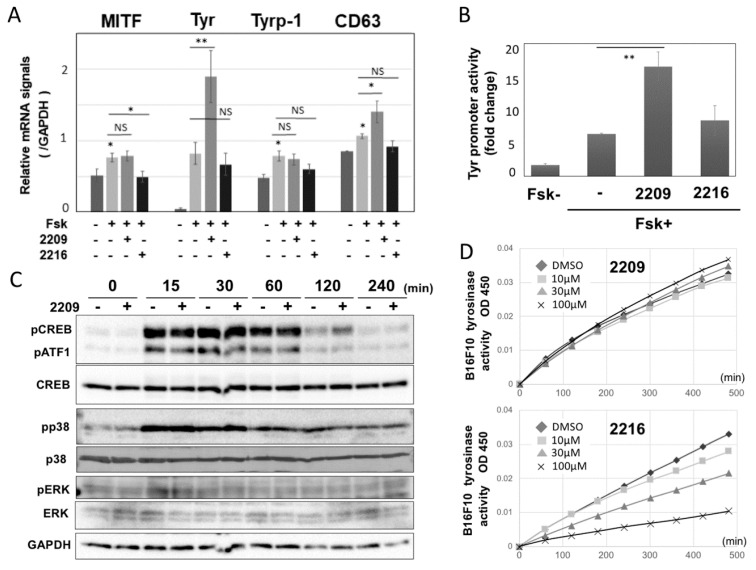
(**A**) B16F10 cells were treated with 10 µM of GIF-2209 or GIF-2216 and 20 µM Fsk for 24 h. The cells were harvested to purify total RNA. The mRNA levels for MITF, TYR, TYRP-1, CD63, and GAPDH were examined by quantitative PCR. Means and S.D. are indicated (*n* = 3). *: *p* < 0.05, ** *p* < 0.01, NS: not significant. (**B**) TYR promoter activity was examined in B16F10 cells treated with GIF-2209 or GIF-2216 for 24 h (*n* = 3). (**C**) Phosphorylation levels of CREB, p38, and ERK in B16F10 cells that had been treated with 20 µM of Fsk and 10 µM of GIF-2209 for indicated periods. (**D**) Tyrosinase activity in cell lysates prepared from Fsk-pretreated B16F10 cells was monitored with l-DOPA (900 µM) in the presence of GIF-2209 or GIF-2216. Means from triplicated experiments are shown.

We examined intracellular signaling events. At 24 h post-treatment, GIF-2209 (10 μM) did not alter the mRNA expression level of MITF, a master transcription factor for melanogenesis, beyond the increase induced by Fsk alone (Figure 3A). This was also the case with TYRP-1 mRNA levels. However, GIF-2209 significantly upregulated TYR and CD63 mRNA levels. In contrast, 10 μM GIF-2216 showed no significant or suppressive effects on TYR, MITF, TYRP-1, or CD63 mRNA levels. The TYR gene promoter activity also supported that GIF-2209 upregulated TYR gene transcription, but GIF-2216 showed no effect on the promoter (Figure 3B).

Since GIF-2209 did not alter the mRNA expression of cAMP-induced MITF, the status of cAMP-dependent signaling molecules was analyzed. The levels of forskolin-induced phosphorylation of CREB, p38, and ERK were not altered by treatment with 10 μM GIF-2209 (Figure 3C). These results suggest that GIF-2209 modulates melanogenesis at the transcriptional level, which may be independent of cAMP-MITF signaling.

To examine whether these compounds modulated TYR enzyme activity, Fsk-pretreated B16F10 cell lysates were analyzed using l-Dopa as the substrate for tyrosinase enzyme (DOPA oxidase activity). GIF-2209 showed no effect on TYR activity, bud GIF-2216 inhibited TYR activity in a concentration-dependent manner (Figure 3D). The same results were obtained, when mushroom TYR enzyme was used (see Materials and Methods). Since GIF-2216 might have an extra action on the TYR enzyme (possibly due to the phenolic structure that is shared with the TYR substrates l-tyrosine and l-Dopa), we decided to focus on only GIF-2209 in subsequent analyses.

### 2.4. GIF-2209 Segregates TYR Containing Vesicles from Those of TYRP-1

**Figure 4:**
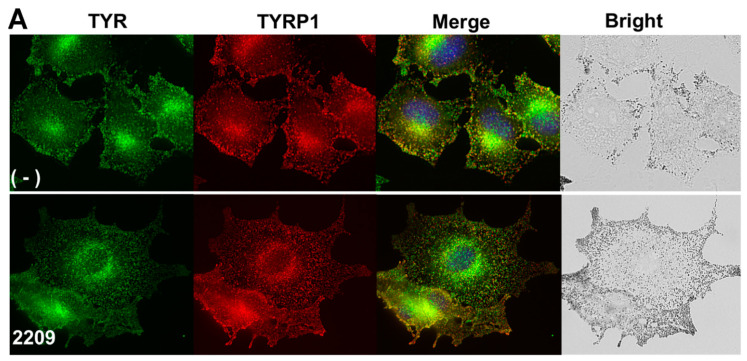

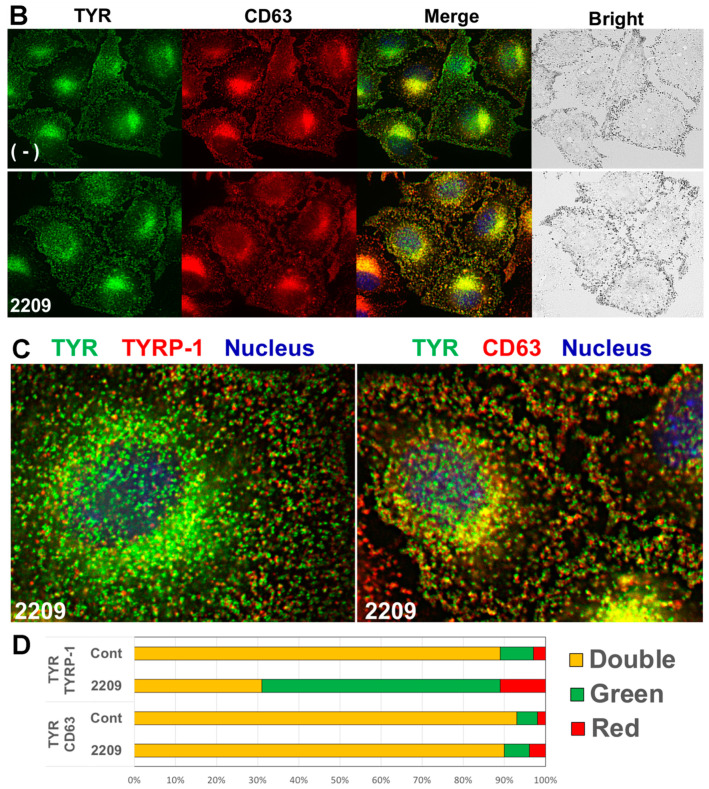
B16F10 cells whose melanogenesis had been initiated by 20 μM of Fsk for 24 h were further treated with or without GIF-2209 (10 μM) for 24 h. Then, the cells were stained with anti-TYR (green) and anti-TYRP-1 (red) antibodies (**A**) or an-ti-CD63 (red) antibodies (**B**). Digital magnification images are shown in (**C**). (**D**) Twenty cells from each staining were selected, and 100 vesicles in each cell were classified into three categories: double positive (orange) or single positive (green or red). Statistical significance (*p* < 0.05, Student’s *t*-test) between control and GIF-2209-treated cells was observed for double-positive (TYR and TYRP-1) spots and TYR single positive spots in cells.

Since GIF-2209 differentially regulated the protein levels of TYR and TYRP-1, we examined the intracellular distribution of TYR and TYRP-1 using immunohistochemical analyses in B16F10 cells. In control cells, signals for TYR and TYRP-1 vesicles almost entirely overlapped (Figure 4A). However, when cells were treated with 10 μM GIF-2209, most TYR signals were segregated from those of TYRP-1 (digitally magnified in Figure 4C).

In contrast to TYRP-1, the protein levels of CD63 were highly correlated with those of TYR (Figure 2). Therefore, intracellular localization of CD63 was examined. As shown in Figure 4B,C, CD63 signals almost entirely overlapped with those of TYR, and were not affected by treatment with GIF-2209 (Figure 4D). These results suggest that TYR and CD63 colocalize with the same intracellular vesicles.

### 2.5. GIF-2209 Stimulates Secretion of mELANOSOMes with High Amounts of TYR, TYRP-1, and CD63

**Figure 5:**
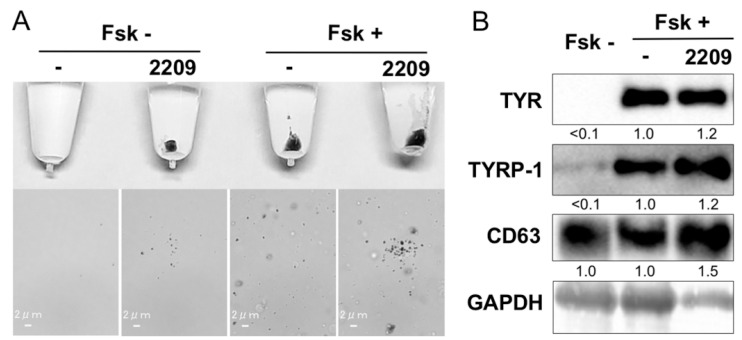
(**A**) B16F10 cells were treated with GIF-2209 (10 μM) in the presence of Fsk (20 μM) for 24 h. The cells were recovered, and melanosomes were precipitated by centrifugation. *n* = 3. (**B**) The melanosome lysate (10 μg) was used for western blot analysis. Signal intensity was normalized to that of GAPDH. The images are representative of triplicate experiments.

Next, we investigated whether GIF-2209 modulated melanosome secretion. Melanosomes in culture medium (50 mL) were recovered by centrifugation (12,000× *g*) and observed by microscopy. As shown in Figure 5A, treatment with 10 μM GIF-2209 accelerated melanosome secretion. Western blot analyses showed that the protein levels of both TYRP-1 and CD63 were higher in melanosomes derived from GIF-2209-treated B16F10 melanoma cells (Figure 5B). These results suggest that the selective secretion of TYRP-1 and CD63 protein through melanosomes might account for the lower content of TYRP-1 and CD63 protein in B16F10 cells treated with 10–30 µM GIF-2209 (Figure 2). Unexpectedly, we found GAPDH in the melanosome lysate. Therefore, we used the band intensity of GAPDH for normalization.

### 2.6. GIF-2209 Modulates the Function and Intracellular Distribution of Lysosomes

**Figure 6:**
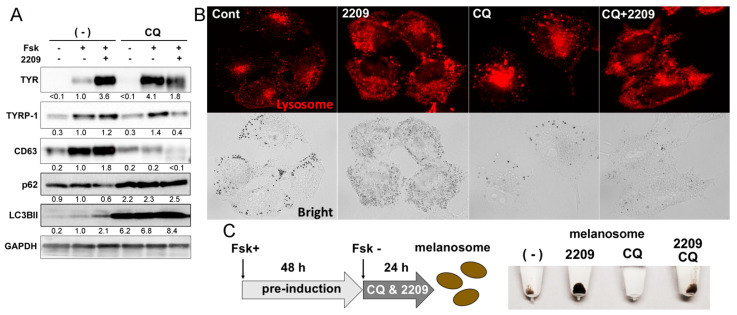
(**A**) B16F10 cells were treated with GIF-2209 (3 μM) and chloroquine (10 μM) in the presence of Fsk (20 μM) for 24 h. The cells were harvested for western blot analysis. The images are representative of triplicate experiments. Signal intensity was normalized to that of GAPDH. (**B**) The lysosomes in cells that had been treated as in (**A**) were stained with Cell Navigator^®^ Lysosome Red. (**C**) Melanogenesis in B16F10 cells was induced by treatment with Fsk (20 μM) for 48 h. Then, the cells were cultured with fresh media containing GIF-2209 (3 μM) and chloroquine (10 μM) for 24 h for melanosome recovery. Signal intensity of each protein was normalized to that of GAPDH.

Lysosomes, marked by CD63, play an important role in melanogenesis. Therefore, we monitored the protein levels of melanogenic enzymes in the presence of the lysosome inhibitor chloroquine [8]. In the presence of 10 µM GIF-2209 and chloroquine, most cells were impaired (rounded) by 6 h. To alleviate cell impairment, the GIF-2209 concentration was decreased to 3 μM, and the incubation was shortened (24 h). When B16F10 cells were treated with chloroquine, the signal intensity of Fsk-induced TYR protein levels increased (Figure 6A). Unexpectedly, co-treatment with chloroquine and GIF-2209 resulted in lower levels of TYR protein. This was also the case for TYRP-1 and CD63. Chloroquine treatment suppressed Fsk-induced upregulation CD63 protein.

To determine the effect of GIF-2209 on other lysosomal functions, we monitored autophagy markers. GIF-2209 treatment downregulated p62 protein level, which is degraded in the process of autophagy, and upregulated LC3BII protein level, which is an autophagy inducer (Figure 6A), suggesting that GIF-2209 might be an autophagy accelerator. In addition, the fluctuations in p62 and LC3BII protein levels were completely abolished by chloroquine (under dysfunction of autophagy systems). Therefore, we surmised that lysosomes might not only degrade melanogenic proteins (TYR and TYRP-1), but also work for maintenance of proper levels of these proteins, and, in some cases, upregulate them.

We examined the intracellular distribution of lysosomes using the lysosome tracer Cell Navigator Lysosome Red (Figure 6B) [20]. In control cells, lysosomes were mainly localized in the perinuclear region. In cells treated with GIF-2209, lysosomes expanded and distributed to all areas of the cytoplasm. Treatment with chloroquine also expanded the size of lysosomes and suppressed Fsk-induced melanogenesis. Chloroquine did not alter the behavior of lysosomes in the presence of GIF-2209. These results suggest that GIF-2209 might modulate lysosomal functions in a manner independent from that of chloroquine.

Finally, we investigated the impact of lysosome disruption on GIF-2209-mediated melanosome secretion. Since chloroquine inhibited melanogenesis, it was difficult to evaluate melanosome secretion under disrupted lysosome conditions (Figure 6A,B). Therefore, melanogenesis was induced by Fsk for 48 h, then, melanosome secretion was evaluated in the absence and presence of chloroquine. GIF-2209 treatment enhanced melanosome secretion, while chloroquine treatment suppressed melanosome secretion even in cells stimulated with GIF-2209, suggesting that GIF-2209 upregulates melanosome secretion via lysosomal pathways.

## 3. Discussion

**Figure 7:**
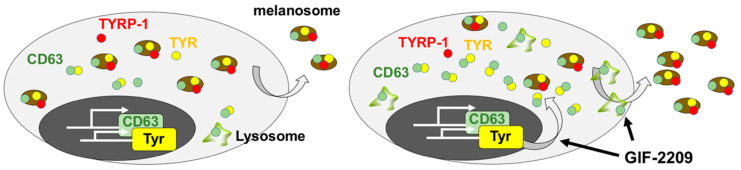
A hypothetical model of GIF-2209-mediated melanogenesis activation. By interacting with lysosome and autophagy systems, GIF-2209 may enhance secretion of matured melanosomes, leading to a lack of melanosomes with melanogenic enzymes in melanocytes. Thus, a high concentration of GIF-2209 ultimately decreased intracellular melanosomes, accompanied by decreased levels of intracellular melanin and melanogenic enzymes including TYR and TYRP-1. In contrast, a lower concentration of GIF-2209 stimulates TYR expression, supplying TYR protein whose level is a rate-limiting step in melanin synthesis, leading to a high content of intracellular melanin.

Here, we report that GIF-2209 has multiple effects on melanogenesis, transcriptional regulation, lysosome modification, and melanosome secretion. In addition, GIF-2209 induced intracellular segregation of tyrosinase-containing vesicles from TYRP-1 vesicles. However, this could be caused by imbalance between the supply of tyrosinase protein and the excretion of TYRP-1 protein (Figure 7).

Skin is an autoregulatory organ in which cells and their physiological functions are maintained by microenvironments (intracellular, autocrine, and percaline systems) [21]. When UV irradiation makes damages in keratinocytes, they produce pro-opiomelanocortin which is processed to stress hormones, adrenocortical hormone, alpha-melanocyte stimulation hormone (α-MSH), and β-endorphin [22]. These hormones regulate skin pigmentation via melanogenesis and contribute to the protection of the skin from UV damages by cooperation with other hormones, prostaglandins, leukotrienes, endothelin 1/3, histamine, stem cell factor, estrogens, androgens, vitamin D3, or bone morphogenic proteins). In addition to the topical regulation, systemic regulations moderate the autoregulatory systems for the skin, in which neuroendocrine systems also play an important role [21].

α-MSH secreted from keratinocytes binds to the melanocortin 1 receptor on the plasma membrane of melanocytes, which acts as an inducer of melanogenesis [1,2,21]. In active melanocytes, melanogenic programs upregulate the transcription of genes whose products contribute to melanin synthesis as well as cellular transport systems. Melanogenic enzymes, such as TYR and TYRP-1, are transported to melanosomes and engaged in melanin synthesis, leading to melanosome maturation. Then, the mature melanosomes are transported to keratinocytes. In hair follicles, hair cycle regulatory systems also contribute to the melanin synthesis, transportation systems, melanosome maturation, and their transfer [23]. Aging also affects the activity and survival of follicular melanocytes.

Unlike humans, mouse skin is poor in melanocytes [21]. In this study, we used the B16F10 mouse melanoma model in which overall systems on melanogenesis may share with those in normal melanocytes in the skin and hair follicles. For example, the enzyme TYR catalyzes the hydroxylation of l-tyrosine to l-DOPA, followed by the catalytic oxidation of l-DOPA to dopaquinone. l-tyrosine and l-DOPA are thus recognized as substrates or intermediates for melanogenesis, but they have also been shown to be biological regulators that can act as positive regulators of melanogenesis in a species-, cell-, genotype-, and environment-dependent manner [24].

Therefore, TYR activity is thought to be correlated with the rate of melanin synthesis. However, some evidences show that TYR activity in cell homogenates does not correlate with the cellular melanin content [25]. It has been proposed that substantial amount of TYR protein is miss-localized to melanosome and trapped in autophagic vacuoles during autophagosome formation. Thus, melanogenesis and autophagy have been believed to link each other, and melanosomes and other lysosome-related organelles share a line of machineries for the transportation of their components [1,2]. However, it is also true that B16F10 cells are melanoma in which a part of autophagy system may be impaired.

Biogenesis of lysosomes in melanocytes is often associated with melanogenesis and melanosome secretion [26]. MITF, a master transcription factor, upregulates the gene expression of melanogenic enzymes and lysosomal factors [27,28]. GIF-2209 specifically upregulated TYR and CD63 mRNA levels without affecting MITF or TYRP-1 mRNA levels. Previously, we reported that ent-11α-hydroxy-15-oxo-kaur-16-en-19-oic acid (11αOH-KA) specifically downregulates the expression of TYR gene, which depends on the MITF promoter binding element [6]. The activation of NF-E2-related factor 2 (NRF2) is a mechanism of 11αOH-KA-mediated suppression of TYR gene expression [29]. However, NRF2 has been reported to suppress MITF gene expression [30,31], leading to the downregulation of TYR and TYRP-1. There evidences suggest that in addition to NRF2, an unknown factor associated with MITF in the promoter may contribute to TYR-specific transcription, which may be also the case for GIF-2209.

The transcription factors TFEB/TFE3 (transcription factor EB/E3) are master regulators of lysosomal function and autophagy [32]. These factors share a structure (bHLH-LZ: basic helix loop helix- leucine zipper) and functions (e.g., target genes and lowering lysosomal pH) with MITF. Both TFEB/TFE3 and MITF are regulated by cell growth signaling and modulate cellular energy metabolism via autophagy [32].

SU5205, a GIF-2209 prototype, was developed as an anticancer compound [19]. This compound inhibits VEGFR2 (tyrosine kinase receptor). Tranexamic acid, a VEGFR inhibitor, has been reported to suppress melanogenesis [33]. Therefore, the activation of melanogenesis by SU5205 and GIF-2209 may not be associated with VEGFR2 inhibition. In contrast, rapamycin, an inhibitor of mammalian target of rapamycin (mTOR), has been reported to accelerate melanogenesis and lysosomal functions via the activation of autophagy systems [34]. However, rapamycin also upregulates the expression of TYRP-1 and TYR in melanocytes [35].

Similarly, inhibition of AKT results in activation of forkhead box O3 (FOXO3), leading to the suppression of melanogenesis in melanocytes [36]. However, AKT inhibition has been found to activate the MITF/TEF family of transcription factors in bone mesenchymal stem cells, promoting autophagy in an mTOR-independent manner [37]. This evidence suggests that autophagy systems may regulate melanogenesis both positively and negatively through combinations of different factors. Although GIF-2209 targets are apparently shared with those of the lysosome/autophagy activator, GIF-2209 modulates only TYR and CD63 gene expression. This evidence suggests that the target of GIF-2209 may work downstream of the AKT/mTOR pathway.

The lysosome inhibitor chloroquine [8] resulted in the accumulation of TYR protein comparable with that induced by GIF-2209. However, the combination of chloroquine and GIF-2209 resulted in decreased TYR protein levels, which was accompanied by low TYRP-1 and CD63 protein levels. Although GIF-2209 decreased TYRP-1 protein level in B16F10 cells, no significant difference in TYRP-1 protein levels in secreted melanosomes prepared from GIF-2209-treated cells was observed. These results suggest that GIF-2209 modulates the rate of melanosome secretion through lysosomal activation rather than through protein degradation.

Further studies are needed to identify the targets of GIF-2209 and to understand the relationship between melanogenesis and lysosome activity. In addition, whether the mechanism of action of GIF-2209 is specific to B16F10 mouse melanoma cells or applicable to other cell types, including normal cells (skin or hair follicle melanocytes) or cells from different species (human, mouse, or fish, etc.), must be determined.

## 4. Materials and Methods

### 4.1. Cell Culture

Mouse B16F10 melanoma cells were cultured in Dulbecco’s modified Eagle’s me-dium (high glucose; Sigma-Aldrich, (Darmstadt, Germany) containing 10% fetal bovine serum (FBS) and 0.6% penicillin-streptomycin (WAKO, Osaka, Japan). B16F10 cells were incubated in a humidified chamber with 5% CO_2_ at 37 °C and were transferred to new culture dishes every 2 days via trypsinization.

B16F10 cells were plated onto 6-well dishes at 3.0 × 10⁴ cells/well. After 24 h, they were treated with oxindole derivatives in the presence of 20 µM forskolin (Fsk, Tokyo Kasei, Tokyo, Japan) for an additional 48 h. After the treatment, cells were suspended in phosphate-buffered saline (PBS: WAKO, Osaka, Japan) and collected into 1.5 mL tubes followed by recovery via centrifugation at 4000 rpm for 2 min [38].

B16F10 cells (1 × 10^4^ cells/well) were treated with the indicated compounds for 48 h. Cell viability was measured using the Cell Counting Kit WST-8 (Dojin, Kumamoto, Japan). Methods of the measurement of melanin content is described in [18].

### 4.2. Preparation of Extracellular Melanosomes

B16F10 cells (5 × 10^5^ cells/well) were seeded in 10 cm dishes. GIF-2209 was added to cells at a final concentration of 3–10 µM, and the cells were incubated for 24 h. Then, the contents of each well were transferred into a 15 mL tube and centrifuged at 2000 rpm for 5 min, and supernatants were collected. The supernatants were re-centrifuged at 12,000 rpm for 20 min, and the pellets were washed twice with PBS.

### 4.3. Western Blot

B16F10 cells (2 × 10⁴ cells) were plated in a 12-well plate. Treated cells were collected into 2 mL tubes and lysed with lysis buffer [0.5 M Tris-HCl buffer (pH 6.8), 20% glycerol, 20 mg bromophenol blue, 2 g sodium dodecyl sulfate (SDS)]. The samples were heated at 98 °C for 10 min, lysates were loaded onto a 10% SDS polyacrylamide gel, and proteins were transferred onto polyvinylidene difluoride membranes (Merck, St. Louis, MI, USA). To detect the target protein(s), anti-CREB, anti-phospho-CREB, anti-p38, and an-ti-phospho-p38 antibodies from Cell Signaling Technology Inc. (Danvers, MA, USA) and anti-ERK, anti-phospho-ERK, anti-LC3BII, anti-p62, and anti-GAPDH (glyceraldehyde-3-phosphate dehydrogenase) antibodies from GeneTex (Irvine, CA, USA) were used. Horseradish peroxidase-conjugated anti-rabbit antibody (MB, Aichi, Japan) was used to detect the protein.

### 4.4. Quantitative PCR (qPCR)

Total RNA was extracted using the FAST GENE RNA Premium Kit (Nippon Gene, Tokyo, Japan). cDNA was synthesized from 200 ng of total RNA using the reverse transcriptase Rivetra Ace (TOYOBO, Osaka, Japan). qPCR was conducted using the SYBR-GREEN based qPCR kit Thunderbird (TOYOBO) and the MyiQ qPCR system (BioRad, Japan, Tokyo, Japan). Primers for TYR, TYRP-1, MITF, and GAPDH are de-scribed in [8]. The primers for CD63 were as follows: Forward: 5′-GCTGTT-GCCTGTGGTCATCA-3′, Reverse: 5′-CTGACTTCACCTGGTCTCTAA-3′.

### 4.5. Immunocytochemistry

B16F10 (3 × 10⁴) cells, seeded onto a poly l-Lysin-coated 18-mm slide glass, were treated with 10 µM GIF-2209 for 24–48 h. Cells were fixed with 4% paraformaldehyde and 0.025% Triton-X 100 (Nacalai Tesque, Kyoto, Japan). After blocking, cells were incubated with the primary antibody (anti-TYR antibody as described in [8], anti-TYRP-1 antibody was from Santa Cruz Biotechnology, Dallas, TX, USA, and anti-CD63 antibody was from MBL) (1/5000), then the secondary antibody (goat anti-rabbit IgG H&L [Alexa Fluor^®^ 488] and goat anti-mouse IgG H&L [Alexa Fluor^®^ 594], Abcam, Pleasanton, CA, USA). Nuclei were stained with 4′,6-diamidino-2-phenylindole (DAPI, ANASPEC INC., CA, USA). Finally, cells were placed on a glass plate. Cell Navigator^®^ Lysosome Staining Kit Red Fluorescence was acquired from ATT Bioquest (Sunnyvale, CA, USA), and the cells were stained according to the manufacturer’s protocol.

### 4.6. Luciferase-Reporter Assay

To assay tyrosinase promoter activity, B16F10 cells (5 × 10⁴) were plated onto a 24-well dish and transformed with pGL4-hRL (100 ng) and pTAL-luc or pTAL mTyr-500 [6] (300 ng each) (Promega, Madison, WI, USA). The plasmids were transfected into cells using Lipofectamine^®^2000 (Thermo Fisher Scientific, Waltham, MA, USA).

### 4.7. Tyrosinase Activity

**Figure 8:**
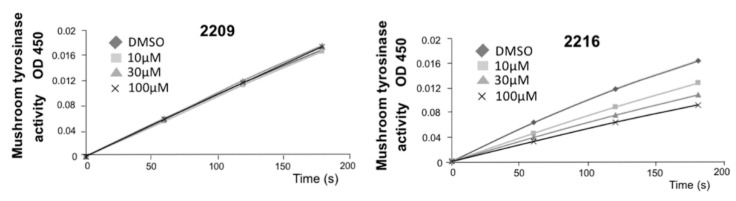
The reliability of the tyrosinase assay system was verified with mushroom tyrosinase (4.15 U).

B16F10 cells were treated with Fsk (20 µM) for 48 h and recovered in 2 mL tubes (approximately 5 × 10^6^ cells). The cells were suspended in 500 µL of PBS (50 mM, pH 6.8) and lysed by sonication for 10 s. Ten µL of the cell lysate with 83 µL of PBS and 2 µL of GIF-2209 or GIF-2216 in DMSO (maximum 100 µM) were mixed in 96-well plates [18]. The reaction was initiated by the addition of l-DOPA (5 µL, final concentration 900 µM), and the monitored using a spectrophotometer (GloMax-Multi Detection System, Promega) at OD450. The reliability of the assay was verified with mushroom tyrosinase (4.15U; Merck) in PBS (Figure 8).

### 4.8. Colocalization Experiments

**Figure 9:**
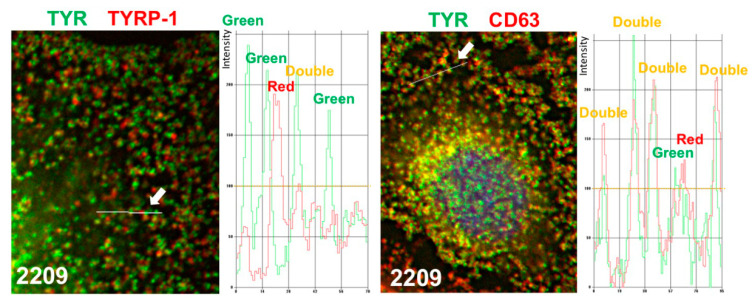
Examples of the categorization of spots (vesicles) into three groups, double positive and single positive (green or red).

Microscopy images were sharpened by a deconvolution program provided by Keyence (Osaka, Japan) with set parameters (sharpness: 18, brightness: 2, elimination: 20%). The intensity of each signal was measured by the line tool and plotted as arbitrary intensity units. Spots (vesicles) with intensity greater than 100 were categorized into three groups: double-positive, green, and red. Examples are shown in Figure 9. We randomly selected twenty cells with flat shapes, and 100 spots (vesicles) in each cell were categorized.

### 4.9. Statistical Analysis

One-way analysis of variance (ANOVA) was used to analyzed data, and Student’s *t*-test was used to compare data from the control (Fsk alone) and treated groups (GIF-2209 or GIF-2216). Bars indicates means and standard deviation.

### 4.10. GIF Compounds

The compounds tested were synthesized by the Knoevenagel condensation of indolin-2-one with substituted benzaldehydes according to the procedure given in the literatures [39,40,41]. The structures of the compounds were confirmed by comparison of the NMR spectral data with reported values.

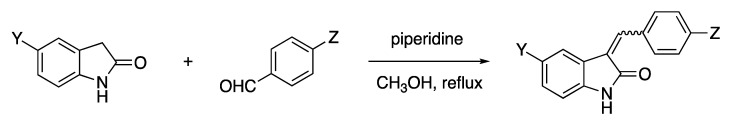

**GIF-2204 (*E*)-3-(2-methoxybenzylidene)indolin-2-one**: ^1^H NMR (400 MHz, DMSO-*d*6) *d* = 3.86 (s, 3H, OCH_3_), 6.83 (t, *J* = 7.1 Hz, 1H, ArH), 6.86 (d, *J* = 7.7 Hz, 1H, ArH), 7.08 (t, *J* = 7.7 Hz, 1H, ArH), 7.16 (d, *J* = 8.3 Hz, 1H, ArH), 7.21 (t, *J* = 7.7 Hz, 1H, ArH), 7.40 (d, *J* = 7.7 Hz, 1H, ArH), 7.49 (t, *J* = 8.3 Hz, 1H, ArH), 7.65 (s, 1H, vinyl), 7.68 (d, *J* = 7.1 Hz, 1H, ArH), 10.6 (br s, 1H, NH).
**GIF-2205 (*E*)-3-(4-chlorobenzylidene)indolin-2-one**: ^1^H NMR (400 MHz, DMSO-*d*6) *d* = 6.86 (t, *J* = 7.6 Hz, 1H, ArH), 6.88 (d, *J* = 7.8 Hz, 1H, ArH), 7.24 (t, *J* = 7.8 Hz, 1H, ArH), 7.48 (d, *J* = 7.8 Hz, 1H, ArH), 7.59 (d, *J* = 8.7 Hz, 2H, ArH), 7.59 (s, 1H, vinyl), 7.73 (d, *J* = 8.7 Hz, 2H, ArH, H-2′,6′), 10.6 (br s, 1H, NH).
**GIF-2206 (*E*)-N,N-dimethyl-4-((2-oxoindolin-3-ylidene)methyl)benzamide**
**(*Z*)-N,N-dimethyl-4-((2-oxoindolin-3-ylidene)methyl)benzamide** (*E*/*Z* mixture, 3.4/1): ^1^H NMR (400 MHz, DMSO-*d*6) *d* = 2.96 and 3.01 (s, 6H, NMe_2_), 6.83 (d, *J* = 7.3 Hz, 1H, ArH, *Z*-isomer) and 6.88 (d, *J* = 7.8 Hz, 1H, ArH, *E*-isomer), 6.86 (t, *J* = 7.8 Hz, 1H, ArH, *E*-isomer) and 7.00 (t, *J* = 7.3 Hz, 1H, ArH, *Z*-isomer), 7.23 (t, *J* = 7.3 Hz, 1H, ArH, *Z*-isomer) and 7.25 (t, *J* = 7.8 Hz, 1H, ArH, *E*-isomer), 7.48 (d, *J* = 8.3 Hz, 1H, ArH), 7.5–7.57 (complex, 3H, ArH), 7.63 (s, 1H, vinyl), 7.76 (d, *J* = 8.3 Hz, 2H, H-2′,6′, ArH, *E*-isomer), 8.40 (d, *J* = 8.3 Hz, 2H, ArH, H-2′,6′, *Z*-isomer), 10.64 and 10.66 (s, 1H, NH, *E*- and *Z*-isomers). ^13^C NMR (100 MHz, DMSO-*d*6) *d* = ~40 (buried under the DMSO signal), 109.97 and 110.77, 120.57 and 121.23, 121.69 and 121.78, 123.04, 127.27 and 127.91, 128.78 and 129.74, 130.97, 132.17, 135.41, 135.9, 137.91, 143.62, 169.06, 170.03.
**GIF-2207 (*E*)-4-((2-oxoindolin-3-ylidene)methyl)benzoic acid**: ^1^H NMR (400 MHz, DMSO-*d*6) *d* = 6.85 (t, *J* = 7.3 Hz, 1H, ArH), 6.88 (d, *J* = 7.8 Hz, 1H, ArH), 7.25 (t, *J* = 7.8 Hz, 1H, ArH), 7.46 (d, *J* = 7.8 Hz, 1H, ArH), 7.65 (s, 1H, vinyl), 7.80 (d, *J* = 8.2 Hz, 2H, ArH), 8.06 (d, *J* = 8.2 Hz, 2H, ArH, H-2′,6′), 10.66 (br s, 1H, NH).
**GIF-2208 (*Z*)-3-(4-(trifluoromethoxy)benzylidene)indolin-2-one**: ^1^H NMR (400 MHz, CDCl_3_) *d* = 6.85 (d, *J* = 7.8 Hz, 1H, ArH), 7.06 (t, *J* = 7.4 Hz, 1H, ArH), 7.25 (t, *J* = 7.8 Hz, 1H, ArH), 7.27 (2H, ArH), 7.50 (s, 1H, vinyl), 7.53 (d, *J* = 7.4 Hz 1H, ArH), 7.73 (br, 1H, NH), 8.32 (d, *J* = 9.2 Hz, 2H, ArH, H-2′,6′). ^13^C NMR (100 MHz, DMSO-*d*6) *d* = 110.00, 120.61, 120.95, 121.71, 125.13, 128.13, 129.89, 131.96, 133.67, 134.38, 135.34, 141.50, 149.65, 167.56.
**GIF-2209 (*E*)-3-(4-(trifluoromethoxy)benzylidene)indolin-2-one**: ^1^H NMR (400 MHz, CDCl_3_) *d* = 6.90 (t, *J* = 7.6 Hz, 1H, ArH), 6.92 (d, *J* = 7.8 Hz, 1H, ArH), 7.24 (t, *J* = 7.6 Hz, 1H, ArH), 7.32 (d, *J* = 8.2 Hz, 2H, ArH), 7.57 (d, *J* = 7.8 Hz, 1H, ArH), 7.70 (d, *J* = 8.2 Hz, 2H, ArH, H-2′,6′), 7.77 (s, 1H, vinyl), 8.49 (br, 1H, NH). ^13^C NMR (100 MHz, DMSO-*d*6) *d* = 110.78, 121.11, 121.74, 121.81, 122.98, 128.92, 131.03, 131.96, 134.26, 134.58, 143.66, 149.27, 168.96.
**GIF-2213 (*E*)-5-chloro-3-(4-(trifluoromethoxy)benzylidene)indolin-2-one**
**(*Z*)-5-chloro-3-(4-(trifluoromethoxy)benzylidene)indolin-2-one** (*E*/*Z* mixture, 3.3/1): ^1^H NMR (400 MHz, DMSO-*d*6) *d* = 6.84 (d, *J* = 8.2 Hz, 1H, ArH, *Z*-isomer) and 6.90 (d, *J* = 9.2 Hz, 1H, ArH, *E*-isomer), 7.25–7.35 (complex, 2H, ArH), 7.47 (d, *J* = 8.7 Hz, 2H, ArH, *Z*-isomer) and 7.55 (d, *J* = 8.7 Hz, 2H, ArH, *E*-isomer), 7.72 (s, 1H, vinyl), 7.83 (d, *J* = 8.7 Hz, 2H, ArH, H-2′,6′, *E*-isomer) and 8.48 (d, *J* = 8.7 Hz, 2H, ArH, H-2′,6′, *Z*-isomer), 7.98 (s, 1H, vinyl), 10.8 (br, 1H, NH). ^13^C NMR (100 MHz, DMSO-*d*6) *d* = 111.38, 112.17, 119.23, 120.67, 120.99, 121.84, 122.40, 122.76, 125.54, 126.05, 127.06, 127.09, 128.18, 129.23, 130.49, 131.94, 133.40, 133.89, 134.67, 136.55, 137.33, 140.13, 142.43, 149.54, 149.97, 167.33, 168.61.
**GIF-2214 (*E*)-3-benzylideneindolin-2-one**: ^1^H NMR (400 MHz, DMSO-*d*6) *d* = 6.85 (t, *J* = 8.4 Hz, 1H, ArH), 6.88 (d, *J* = 7.8 Hz, 1H, ArH), 7.23 (t, *J* = 7.8 Hz, 1H, ArH), 7.45–7.56 (complex, 4H, ArH), 7.63 (s, 1H, vinyl), 7.70 (d, *J* = 7.3 Hz, 2H, ArH, H-2′,6′), 10.61 (br s, 1H, NH).
**GIF-2215 (*E*)-5-nitro-3-(4-(trifluoromethoxy)benzylidene)indolin-2-one**
**(*Z*)-5-nitro-3-(4-(trifluoromethoxy)benzylidene)indolin-2-one** (*E*/*Z* mixture, 1/2.3): ^1^H NMR (400 MHz, DMSO-*d*6) *d* = 7.03 (d, *J* = 8.7 Hz, 1H, ArH, *Z*-isomer) and 7.08 (d, *J* = 9.2 Hz, 1H, ArH, *E*-isomer), 7.51 (d, *J* = 8.5 Hz, 2H, ArH, *Z*-isomer) and 7.58 (d, *J* = 7.8 Hz, 2H, ArH, *E*-isomer), 7.87 (s, 1H, vinyl, *E*-isomer), 7.91 (d, *J* = 8.7 Hz, 1H, ArH, *Z*-isomer), 8.17–8.24 (complex, 4H, ArH, *Z*-isomer), 8.28 (s, 1H, vinyl, *E*-isomer), 8.54 (d, *J* = 8.5 Hz, 2H, ArH, H-2′,6′, *Z*-isomer), 8.72 (s, 1H, ArH, *Z*-isomer), 11.39 (br s, 1H, NH). ^13^C NMR (100 MHz, DMSO-*d*6) *d* = 110.05, 110.78, 116.46, 118.05, 121.04, 121.92, 125.92, 126.17, 127.3, 132.14, 133.20, 134.97, 138.23, 139.30, 142.09, 142.66, 146.88, 149.2, 150.29, 167.80, 169.13.
**GIF-2216 (*E*)-3-(4-hydroxybenzylidene)indolin-2-one**: ^1^H NMR (400 MHz, DMSO-*d*6) *d* = 6.87 (dt, *J* = 2.7 and 7.8 Hz, 1H, ArH), 6.90 (d, *J* = 8.5 Hz, 2H, ArH), 7.21 (t, *J* = 7.6 Hz, 1H, ArH), 7.53 (s, 1H, vinyl), 7.61 (d, *J* = 8.5 Hz, 2H, ArH, H-2′,6′), 7.69 (d, *J* = 7.8 Hz, 1H, ArH), 10.52 (br, 1H, NH).

**Figure 10:**
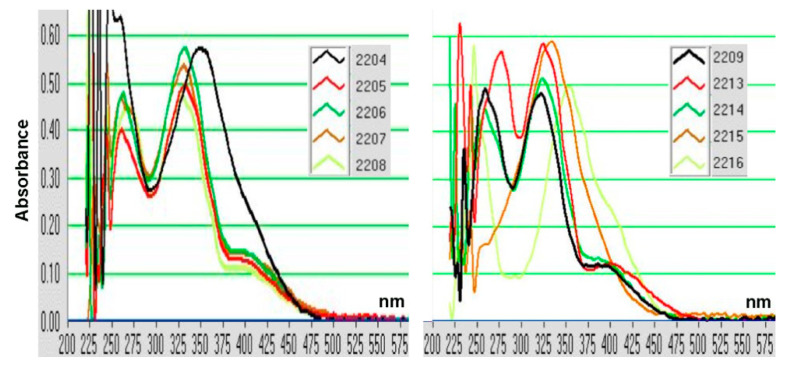
Absorbance spectra of GIF compounds (5 μM in PBS).

Absobance spectra of the compounds are shown in Figure 10.

## 5. Conclusions

The oxindole GIF-2209 is a multifunctional compound that induces expression of TYR and CD63 mRNA and protein in B16F10 cells. GIF-2209 upregulated and downregulated TYR and TYRP-1 protein, respectively, in a concentration-dependent and lysosomal activity-dependent manner. GIF-2209 enhances melanosome secretion, which is associated with the redistribution of lysosomes in the cytoplasm. These results suggest that GIF-2209 could be a new tool for the study of lysosome-related physiological activities.

## Data Availability

The data presented and additional data from this study are available upon request from the corresponding author.

## Sample Availability

Samples of the compounds (GIF-2204-2216) and anti-TYR antibody are available from the authors.
